# Workflow for Accurate Measurement of PDI Reductase Kinetics Using a Fluorescent Disulfide Substrate

**DOI:** 10.17912/micropub.biology.002334

**Published:** 2026-09-08

**Authors:** Nathan Ponzar, Nicola Pozzi

**Affiliations:** 1 Biochemistry and Molecular Biology, Saint Louis University, St Louis, MO, United States

## Abstract

Protein disulfide isomerase (PDI) is essential for oxidative protein folding, proteostasis, and redox regulation and has become a promising target for thromboinflammation, cancer, and neurodegenerative disorders. Fluorogenic disulfide substrates like Bodipy FL L-cystine (BDSS) are essential for mechanistic studies but have low affinity for PDI, necessitating high micromolar concentrations to measure Michaelis-Menten kinetics. These conditions can lead to optical artifacts, requiring development of correction methods to enable rigorous characterization of PDI inhibitors and allosteric modulators. Here, we present a simple workflow for correcting fluorescence quenching during BDSS reduction assays and provide an example of its utility with two allosteric modulators.

**Figure 1. Workflow for correction of steady-state kinetics of BDSS reduction by PDI:** **A.** Initial rates of BDSS reduction in fluorescence show inhibition at high substrate (>20 µM) concentrations. **B.** Series of BDSH standard curves measured in the presence of increasing BDSS concentrations. **C.** Plot of slopes fit with Stern-Volmer model. Error bars show the standard deviation from three titrations. **D.** Rates of BDSS reduction from *A* divided by BDSS-specific standard curves, as in *B,* per enzyme, i.e. *k* = *v*_raw_ /F _n_ [E]. **E.** Rates of BDSS reduction from absorbance at 504, i.e. *k* = *v*_504_ /Δɛ _504_ [E]. **F.** Results of “stop-and-dilute” assay. Each data set represents raw data after correction for the dilution factor indicated to the right of the data set. The 16- and 64-fold diluted data sets satisfactorily fit the Michaelis-Menten equation and display *K*_M_ of 2.1 µM. **G.** Uncorrected fluorescence rates in the presence of 0 compound (black) or 50 µM bepristat 2a (B2a, red) or rutin (Q3R, green). **H.** Correction of rates in G by BDSS-specific slopes as in *C* , fit with Michaelis-Menten equation.

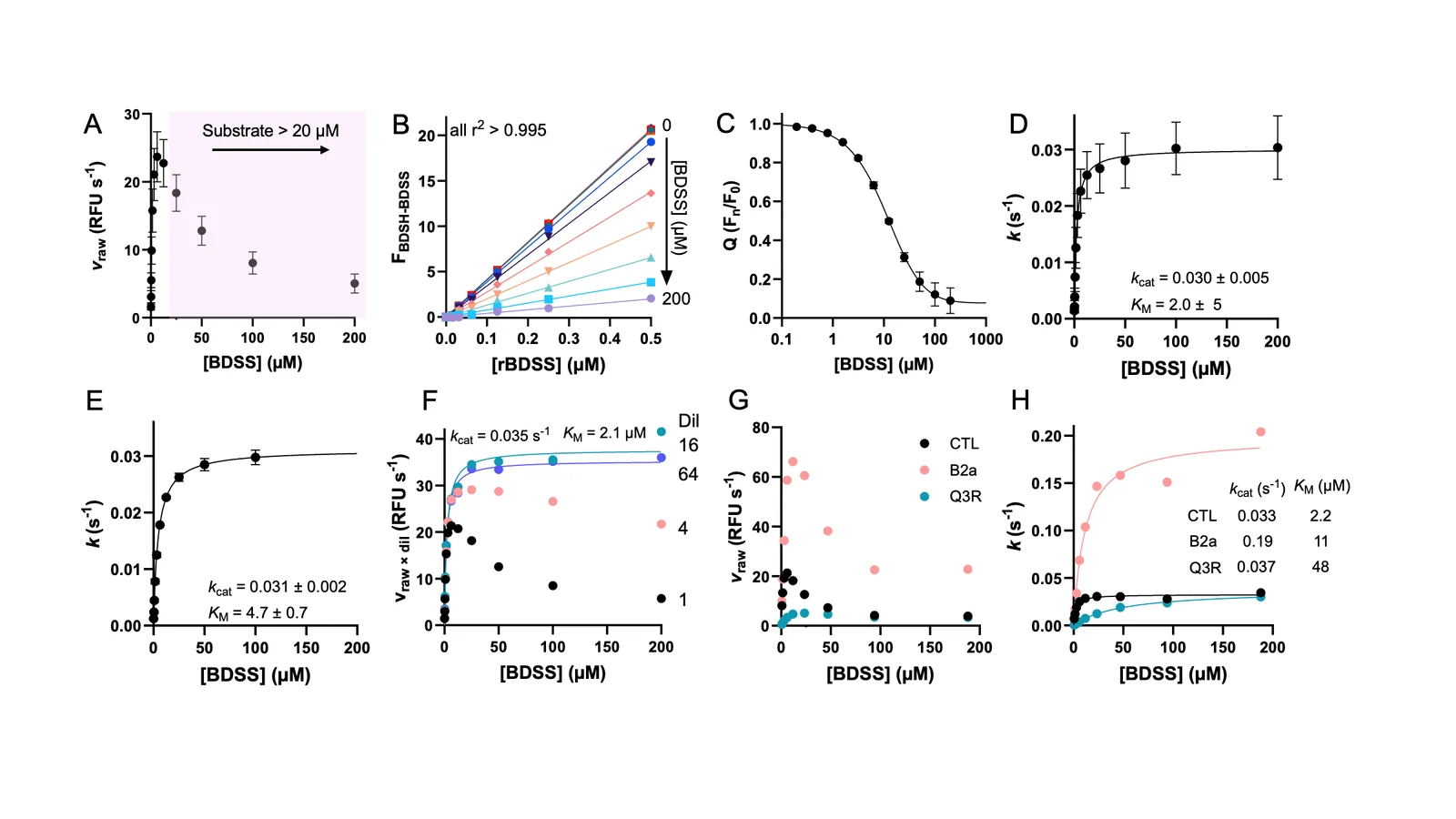



## Description

Protein disulfide isomerase (PDI) is the most prominent member of the thiol isomerase (TI) protein family, which includes about 20 members in humans (Ali Khan & Mutus, 2014). Through its reductase, oxidase, and isomerase activities, PDI facilitates disulfide-bond formation and drives oxidative protein folding within the endoplasmic reticulum (ER) (Oliveira et al., 2026). Beyond the ER, PDI functions in the cytosol, at the cell surface, and in the extracellular space, where it regulates diverse physiological processes (Fu et al., 2020; Grek & Townsend, 2014; Schulman et al., 2015; Tanaka et al., 2020). And increasing evidence that PDI is dysregulated in disease has made it a promising therapeutic target in thrombosis (Essex & Wang, 2024; Flaumenhaft & Furie, 2016; Gaspar & Gibbins, 2021; Sun et al., 2026; Wang et al., 2026; Xiong et al., 2020), autoimmunity (Kumar et al., 2021; Müller-Calleja et al., 2018; Passam et al., 2010), cancer (Luhle et al., 2026; Nie et al., 2025; Zhang et al., 2026), and other diseases (Puhl et al., 2023; Rasi et al., 2024; Rawarak et al., 2019).

Interest in targeting PDI has led to the development of experimental assays to assess its activity. Drug screening and characterization have traditionally employed an insulin reduction assay that relies on the aggregation and turbidity of reduced insulin solutions as a readout of PDI activity (Smith et al., 2004). More recently, small fluorescent disulfide-bonded substrates have become more commonly used because the fluorescence generated upon reduction can be directly converted to rates of PDI turnover, providing a more quantitative approach that enables mechanistic studies (Ponzar et al., 2025; Raturi et al., 2005). Bodipy FL L-cystine (BDSS) is a commonly used substrate. It consists of two disulfide-bonded cysteine molecules, each labeled with a bodipy fluorophore (Foster & Thorpe, 2017). In the disulfide state, when excited at 470 nm, bodipy is highly quenched. However, cleavage of the disulfide bond by PDI results in the formation of the fully reduced product, Bodipy FL cysteine (BDSH), which exhibits ~55-fold higher fluorescence than intact BDSS (Foster & Thorpe, 2017).

Previous studies, including our own (Ponzar et al., 2025), typically employed BDSS concentrations up to 20 μM. Under these conditions, in a standard 96-well plate format, reaction rates approach apparent saturation and follow a classical Michaelis-Menten relationship, enabling estimation of *K _M_* and *k _cat_* . However, extending substrate concentrations beyond 20 μM revealed an unexpected second phase in which apparent reaction rates declined ( **Fig. 1A** ). Two explanations were considered: substrate inhibition and optical quenching. Because absorbance increases with substrate concentration, we hypothesized that optical effects were responsible. To test this possibility, we examined the influence of intact BDSS on the fluorescence of fully reduced BDSH. Three observations supported a quenching mechanism. First, BDSH fluorescence decreased progressively as BDSS concentrations increased from 0 to 200 μM ( **Fig. 1B** ). Second, the relationship between molar fluorescence and BDSS concentration followed a hyperbolic trend that was accurately described by the Stern-Volmer equation ( **Fig. 1C** ) (Lakowicz, 2006). Third, converting raw fluorescence rates into molar rates using BDSH calibration curves generated at each BDSS concentration eliminated the apparent inhibitory phase and restored a near-ideal Michaelis-Menten profile, yielding a *k _cat_* of 0.030 s ^-1^ and *K _M_* of 2.0 µM ( **Fig. 1D** ). Together, these findings identify optical quenching, rather than substrate inhibition, as the primary cause of the apparent loss of activity at elevated substrate concentrations.

To independently validate this conclusion, we quantified BDSS reduction using absorbance measurements. Similar to fluorescence, reduction of BDSS results in an increase in absorbance at 504 nm; however, unlike fluorescence, the change is modest (approximately 2-fold) and necessitates higher enzyme concentrations due to a lower signal-to-noise ratio to accurately determine rates. Moreover, substrate concentrations are limited to below 100 µM because higher concentrations violate the linearity of the Lambert-Beer law. Despite these constraints, absorbance measurements produced a satisfactory Michaelis-Menten profile with a *k _cat_* of 0.031 s⁻¹ and a *K _M_* of 4.7 μM ( **Fig. 1D** ). The close agreement in *k _cat_* values was encouraging, but the apparent *K _M_* suggested that higher enzyme concentrations artificially inflate the apparent affinity. To address this possibility, we developed an independent stop-and-dilute protocol in which reactions were quenched with iodoacetamide and subsequently diluted until concentration-dependent fluorescence effects were eliminated ( **Fig. 1E** ). This approach yielded a *K _M_* of 2.1 μM and a comparable *k _cat_* after correction for dilution, closely matching the values obtained with the continuous fluorescence-based measurements in **Fig. 1D** . These orthogonal approaches confirm that optical quenching, rather than substrate inhibition, underlies the apparent decline in activity at high BDSS concentrations and validate an empirical correction strategy based on BDSH standards. Experimental validation is important since optical artifacts are notoriously difficult to correct on theoretical grounds in plate reader detection (Weitner et al., 2022).

We next assessed the utility of this correction method using bep2a and rutin, established PDI allosteric activator and inhibitor, respectively (Bekendam et al., 2016; Lin et al., 2015; Ponzar et al., 2026). Earlier studies performed with BDSS concentrations up to 20 μM showed that bep2a increased *k _cat_* approximately sixfold while also increasing *K _M_* , whereas rutin caused a roughly tenfold increase in *K _M_* accompanied by a moderate reduction in *k _cat_* (Ponzar et al., 2025). Extending substrate concentrations to 200 μM again produced the characteristic decline in apparent rates ( **Fig. 1G** ), but application of the quenching correction restored the expected kinetic behavior ( **Fig. 1H** ). The resulting fits reproduced previously observed trends while refining their quantitative interpretation. Most notably, rutin produced an approximately 20-fold increase in *K _M_* , whereas its effect on *k _cat_* became negligible, with a value similar to that of unbound PDI at saturating substrate concentrations, indicating that the previously reported reduction in *k _cat_* measured at 20 μM BDSS likely reflects an optical artifact rather than true catalytic inhibition. These findings support our model whereby bep2a allosterically activates PDI, whereas rutin allosterically impairs substrate engagement (Ponzar et al., 2025; Ponzar et al., 2026). More broadly, correcting for substrate-dependent quenching expands the usable concentration range of BDSS assays, allowing for more precise quantitative mechanistic characterization of these modulators and opening avenues for rigorous investigation into the structural pharmacology of PDI.

Although this report focuses on BDSS, another widely used fluorescent disulfide substrate for studying PDI is di-eosin GSSG (Di-E-GSSG) (Raturi et al., 2005). Di-E-GSSG consists of oxidized glutathione (GSSG) labeled at both N-termini with eosin (E) fluorophores. Like BDSS, Di-E-GSSG is highly quenched in the oxidized state and increases in fluorescence upon PDI-mediated reduction, a behavior attributed in part to intramolecular homo-FRET between closely positioned eosin dyes (Tang et al., 2021). However, the two substrates differ in both scaffold and fluorophore chemistry. Glutathione is a tripeptide, making Di-E-GSSG much larger than BDSS; in addition, eosin is a negatively charged, polybrominated fluorophore, whereas bodipy is nonpolar and difluorinated. Moreover, the unlabeled forms of both substrates form the basis of important physiological redox buffer systems, with GSSG contributing to intracellular glutathione redox buffering and L-cystine representing an important extracellular cysteine/cystine redox couple (Banerjee, 2012). Thus, BDSS and Di-E-GSSG are best viewed as complementary fluorescent substrates rather than interchangeable assays. Di-E-GSSG has often been used at relatively low substrate concentrations, for example 150 nM, well below the estimated *K _M_* value (Raturi et al., 2005), particularly for screening applications or qualitative comparisons of PDI activity, conditions under which concentration-dependent optical artifacts may be less apparent. BDSS can also be used in this lower-concentration format for similar screening or qualitative applications. However, when the goal is to define full steady-state kinetic behavior or evaluate how PDI ligands alter *K _M_* and *k _cat_* , higher substrate concentrations may be required. Under these conditions, substrate-dependent fluorescence effects become an important consideration for both substrates. Indeed, in our hands, Di-E-GSSG also shows concentration-dependent fluorescence effects at concentrations exceeding 10 µM. Accordingly, assay selection and correction requirements should depend on whether the experiment is intended for qualitative screening or rigorous kinetic analysis, not simply Di-E-GSSG versus BDSS.

## Methods

Bodipy FL L-cystine substrate (BDSS) was purchased as a custom order from MedChemBio and was purified to greater than 95% purity as determined by HPLC and NMR. Human recombinant PDI (aa 18-479) was expressed in *E.coli* and purified by Ni affinity chromatography, followed by SEC in a Superdex S200 GL10/300 column as described before (Chinnaraj et al., 2022; Ponzar et al., 2025; Ponzar et al., 2026). Protein samples were aliquoted under argon and stored at -80°C until used.

Activity assays were conducted in PBS (Sigma-Aldrich) supplemented with 2 mM EDTA at a pH of 7.4 (PBSE) at 25C. Black 96-well plates with NBS treatment were used (CORNING 3991). 60 nM PDI prepared with 20 µM DTT was combined with an equal volume of 2X substrate just before starting the kinetic reads, resulting in a final concentration of 30 nM PDI and 10 µM DTT in a final volume of 220 µl. Final substrate concentrations ranged from 0-200 µM. Control reactions containing 10 µM DTT, but no PDI, were included in every assay for subtraction of the rates of DTT-mediated BDSS reduction. Fluorescence was read at regular intervals with a TECAN Spark plate reader with 470 nm excitation and 515 nm detection, both with 20 nm bandpass filters, and rates of fluorescence increase were determined by linear regression of the data from 200-600 seconds. Depletion of substrate was <10%, fulfilling the definition of initial velocity. For assays in the presence of compounds, a small volume of concentrated stock was added to the 2X protein solution at 40 µM, which was reduced to 20 µM when combined with substrate.

To measure the fluorescence enhancement upon reduction of BDSS to two BDSH molecules, BDSS (0-1 µM) was reduced with a 10-fold excess of DTT until complete conversion of substrate to product, as monitored by a steady fluorescent signal. Intact BDSS was titrated from 0-400 µM, and 110 µl was dispensed into all 96-well plates (CORNING 3991). 110 µl of the pre-reduced BDSH solutions was diluted twofold into the BDSS solutions in the plate to create a matrix of BDSS and BDSH. Fluorescence was read immediately, preventing substantial reduction of BDSS by residual DTT in the BDSH solution. Background fluorescence from the BDSS was subtracted from the BDSH standards’ fluorescence. Fluorescence of reduced standards was plotted as a function of the concentration of reduced BDSS rather than of BDSH so that rates are calculated in terms of enzyme turnover rather than product generated. Slopes for each BDSS concentration in **Fig. 1C** were fit with a modified, empirical form of the Stern-Volmer equations, combining the dynamic and static quenching terms into a single, apparent quenching constant (Lakowicz, 2006).

In the “stop-and-dilute” assay, reactions were halted with iodoacetamide (IAM) at each minute from 2 to 10 minutes. Reactions were diluted 4, 16, and 64-fold in PBSE along with an IAM-quenched set of BDSH standards. Rates were determined by linear regression of the fluorescence increase over time of the IAM-quenched reactions. Rates were converted to molar rates by the BDSH standards and then multiplied by the dilution factor. In the absorbance-based assay, Greiner clear 96-well plates were used (655801), and rates of increasing absorbance were converted to molar rates by a standard curve in the absence of BDSS, because absorbance is not subject to artifacts at high concentrations as long as absorbance values remain in the Lambert-Beer’s law regime.

To convert fluorescence signal to reaction rates, we employed the following workflow. 1) Fluorescence from BDSH standard curves was measured in the presence of increasing BDSS concentrations (F _n_ ) and none (F _0_ ), and background BDSS signal was subtracted ( **Fig. 1B** ). 2) Fluorescence of BDSH standards at each BDSS concentration was fit with a line anchored to the origin (Y-intercept = 0) and plotted against concentration in terms of reduced BDSS molecules (rBDSS = BDSH/2) ( **Fig. 1B** ). 3) To convert fluorescence rates to molar rates of substrate cleavage on a per-enzyme basis, raw fluorescence rates ( *v*_raw_ ) were divided by their BDSS-specific standard curve slopes (F _n_ ) and the enzyme concentration (30 nM) ( **Fig. 1D** ). All data fitting was performed in GraphPad Prism 10.6.
